# tna: An R Package for Transition Network Analysis

**DOI:** 10.1177/01466216251348840

**Published:** 2025-06-05

**Authors:** Santtu Tikka, Sonsoles López-Pernas, Mohammed Saqr

**Affiliations:** 1 4168 University of Jyväskylä, Jyväskylä, Finland; 2 163043 University of Eastern Finland, Joensuu, Finland

**Keywords:** tna, transition network analysis, Markov models, sequence analysis, learning analytics, R package, social network analysis, complex dynamic systems

## Abstract

Understanding the dynamics of transitions plays a central role in educational research, informing studies of learning processes, motivation shifts, and social interactions. Transition network analysis (TNA) is a unified framework of probabilistic modeling and network analysis for capturing the temporal and relational aspects of transitions between events or states of interest. We introduce the R package tna that implements procedures for estimating the TNA models, building the transition networks, identifying patterns and communities, computing centrality measures, and visualizing the networks. The package also implements several functions for statistical procedures that can be used to assess differences between groups, stability of centrality measures and importance of specific transitions.

## Description of the Package

The tna package (Saqr et al., 2025e) is designed to implement the complete transition network analysis (TNA, Saqr et al., 2025b, 2025c) pipeline in R (R Core Team, 2024). The transition network models can be constructed directly from a matrix of edge weights and common data formats for representing sequence data, but the package also implements tools to import event data of various formats. Several model types are available, including standard Markov models, frequency-based transition models (Saqr et al., 2025a, 2025d), co-occurrence models, and many others. The main features of the package include finding patterns of transitions, computing centrality measures, community detection, and visualization. Network comparisons can be carried out using various similarity and dissimilarity measures (Tantardini et al., 2019) at the network level or at the edge level. All of these functionalities are also supported for clustered or grouped models (López-Pernas et al., 2025), for which additional features are also available, such as comparisons between groups.

The tna package implements several statistical validation tools to quantify the uncertainty of the network, discover significant transitions, and find differences between distinct groups. For models built from sequence data, non-parametric bootstrapping (Efron & Tibshirani, 1994) can be used to discover strong edges and consequently prune spurious edges from the network, leading to more robust inference and simplified interpretations. To analyze the stability of the centrality measures, case-dropping is implemented (Epskamp et al., 2017). Multiple models can be compared with a permutation test to discover significant differences in the transitions.

## Availability, Documentation, and Distribution

The tna package, documentation, and the supplementary materials are available from the Comprehensive R Archive Network (https://CRAN.R-project.org/package=tna) and GitHub (https://github.com/sonsoleslp/tna). The supplementary materials include complete documentation of the package features and provide illustrative examples and tutorials on the practical use of the package. The documentation and tutorials can also be viewed online on the package website (https://sonsoles.me/tna/). A Shiny application for the tna package is also available online (https://sonsoleslp.shinyapps.io/tnaapp/).

## Supplemental Material

Supplemental Material - tna: An R Package for Transition Network Analysis Supplemental Material for tna: An R Package for Transition Network Analysis by Santtu Tikka, Sonsoles López-Pernas, and Mohammed Saqr in Applied Psychological Measurement
