# Supplementary material for: tna: An R Package for Transition Network Analysis
Source: Appl Psychol Meas. 2025 Jun 5;49(6):326–8. doi: 10.1177/01466216251348840 (PMC12141252; doi:10.1177/01466216251348840)
Supplement: Supplemental Material - tna: An R Package for Transition Network Analysis [file sj-zip-1-apm-10.1177_01466216251348840.zip › tna_0.4.0.tar.gz!/tna/tests/testthat/Rplots.pdf]

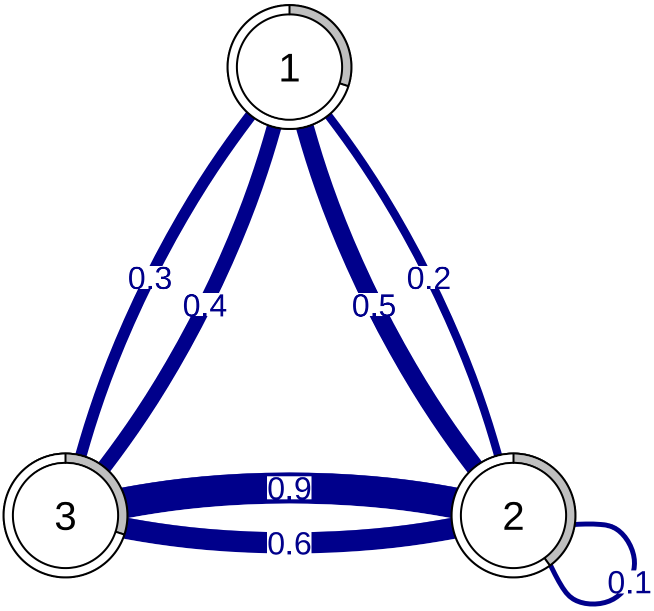
1
2
3
0.3
0.4
0.5
0.2
0.9
0.6
0.1

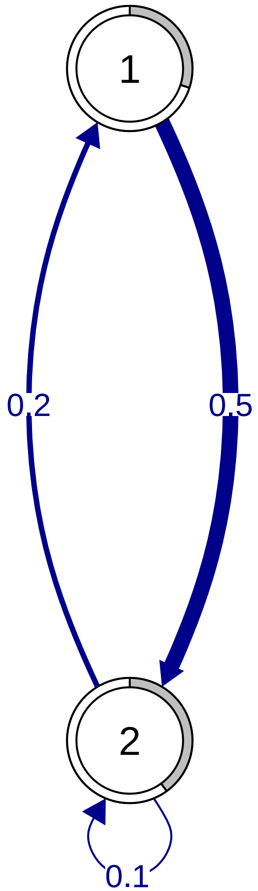
1
0.2
0.5
2
0.1

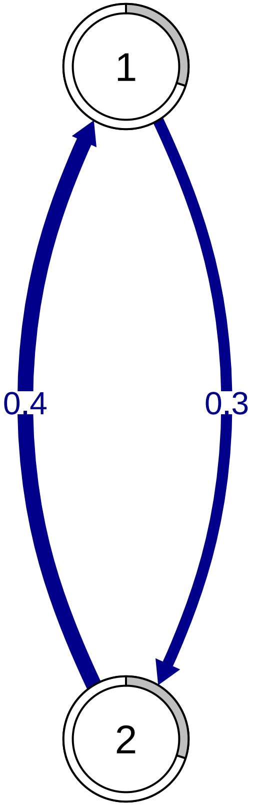
1
2
0.4
0.3

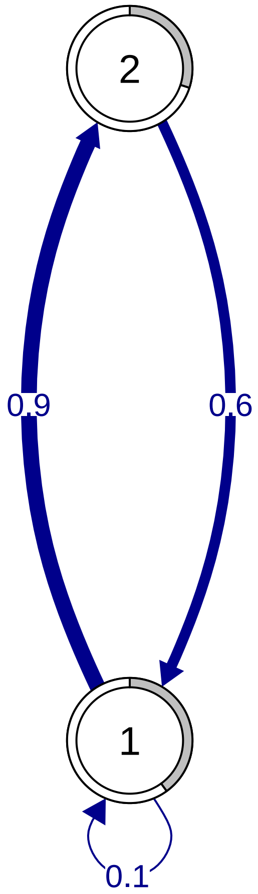
2
0.9
0.6
1
0.1
