# Supplementary material for: tna: An R Package for Transition Network Analysis
Source: Appl Psychol Meas. 2025 Jun 5;49(6):326–8. doi: 10.1177/01466216251348840 (PMC12141252; doi:10.1177/01466216251348840)
Supplement: Supplemental Material - tna: An R Package for Transition Network Analysis [file sj-zip-1-apm-10.1177_01466216251348840.zip › tna_0.4.0.tar.gz!/tna/inst/doc/tna.html]

Getting started with the tna package


# Getting started with the tna package

This vignette showcases some basic usage of the `tna`
package. First we load the package that we will use for this
example.

```
library("tna")
library("tibble")
library("dplyr")
library("gt")
```

We also load the `group_regulation` data available in the
package (see `?group_regulation` for further information)

```
data("group_regulation", package = "tna")
```

We build a TNA model using this data with the `tna()`
function .

```
tna_model <- tna(group_regulation)
```

To visualize the model, we can use the standard `plot()`
function.

```
plot(tna_model, cut = 0.2, minimum = 0.05, 
     edge.label.position= 0.8, edge.label.cex = 0.7)
```

The initial state probabilities are

```
data.frame(`Initial prob.` = tna_model$inits, check.names = FALSE) |>
  rownames_to_column("Action") |>
  arrange(desc(`Initial prob.`)) |>
  gt() |>
  fmt_percent()
```

| Action | Initial prob. |
| --- | --- |
| consensus | 21.40% |
| plan | 20.45% |
| discuss | 17.55% |
| emotion | 15.15% |
| monitor | 14.40% |
| cohesion | 6.05% |
| synthesis | 1.95% |
| coregulate | 1.90% |
| adapt | 1.15% |

and the transitions probabilities are

```
tna_model$weights |>
  data.frame() |>
  rownames_to_column("From\\To") |>
  gt() |>
  fmt_percent()
```

| From\To | adapt | cohesion | consensus | coregulate | discuss | emotion | monitor | plan | synthesis |
| --- | --- | --- | --- | --- | --- | --- | --- | --- | --- |
| adapt | 0.00% | 27.31% | 47.74% | 2.16% | 5.89% | 11.98% | 3.34% | 1.57% | 0.00% |
| cohesion | 0.29% | 2.71% | 49.79% | 11.92% | 5.96% | 11.56% | 3.30% | 14.10% | 0.35% |
| consensus | 0.47% | 1.49% | 8.20% | 18.77% | 18.80% | 7.27% | 4.66% | 39.58% | 0.76% |
| coregulate | 1.62% | 3.60% | 13.45% | 2.34% | 27.36% | 17.21% | 8.63% | 23.91% | 1.88% |
| discuss | 7.14% | 4.76% | 32.12% | 8.43% | 19.49% | 10.58% | 2.23% | 1.16% | 14.10% |
| emotion | 0.25% | 32.53% | 32.04% | 3.42% | 10.19% | 7.68% | 3.63% | 9.98% | 0.28% |
| monitor | 1.12% | 5.58% | 15.91% | 5.79% | 37.54% | 9.07% | 1.81% | 21.56% | 1.61% |
| plan | 0.10% | 2.52% | 29.04% | 1.72% | 6.79% | 14.68% | 7.55% | 37.42% | 0.18% |
| synthesis | 23.47% | 3.37% | 46.63% | 4.45% | 6.29% | 7.06% | 1.23% | 7.52% | 0.00% |

The function `centralities()` can be used to compute
various centrality measures (see `?centralities` for more
information). These measures can also be visualized with the
`plot()` function.

```
centrality_measures <- c("BetweennessRSP", "Closeness", "InStrength", "OutStrength")
cents_withoutloops <- centralities(
  tna_model,
  measures = centrality_measures,
  loops = FALSE,
  normalize = TRUE
)
plot(cents_withoutloops, ncol = 2, model = tna_model)
```
