# Supplementary material for: tna: An R Package for Transition Network Analysis
Source: Appl Psychol Meas. 2025 Jun 5;49(6):326–8. doi: 10.1177/01466216251348840 (PMC12141252; doi:10.1177/01466216251348840)
Supplement: Supplemental Material - tna: An R Package for Transition Network Analysis [file sj-zip-1-apm-10.1177_01466216251348840.zip › tna_0.4.0.tar.gz!/tna/inst/doc/complete_tutorial.html]

A showcase of the main tna functions


# A showcase of the main tna functions

# Tutorial of TNA with R

```
# Install `tna` if you haven't already
# install.packages("tna")
library("tna")
data("group_regulation")
```

## Building `tna` Model

```
model <- tna(group_regulation)
print(model)
```

## Plotting and interpreting `tna` models

### Interpretation of the model

```
# TNA visualization
plot(model, minimum = 0.05, cut = 0.1)
```

### Pruning and retaining edges that “matter”

```
layout(matrix(1:4, ncol = 2, byrow = T))
# Pruning with different methods (using comparable parameters)
pruned_threshold <- prune(model, method = "threshold", threshold = 0.15)
pruned_lowest <- prune(model, method = "lowest", lowest = 0.15)
pruned_disparity <- prune(model, method = "disparity", level = 0.5)

# Plotting for comparison
plot(pruned_threshold)
plot(pruned_lowest)
plot(pruned_disparity)
plot(model, minimum = 0.05, cut = 0.1)
```

## Patterns

```
layout(t(1:2))
# Identify 2-cliques (dyads) from the TNA model, excluding loops in the visualization
# A clique of size 2 is essentially a pair of connected nodes
cliques_of_two <- cliques(
  model, 
  size = 2, 
  threshold = 0.15  # Only consider edges with weight > 0.15
)
print(cliques_of_two)
#> Number of 2-cliques: 2 (weight threshold = 0.15)
#> Showing 2 cliques starting from clique number 1
#> 
#> Clique 1:
#>           consensus plan
#> consensus     0.082 0.40
#> plan          0.290 0.37
#> 
#> Clique 2:
#>           consensus discuss
#> consensus     0.082    0.19
#> discuss       0.321    0.19
plot(cliques_of_two, vsize = 15, edge.label.cex = 2, esize = 20, ask = F)
```

```
layout(t(1:3))
# Identify 3-cliques (triads) from the TNA_Model
# A clique of size 3 means a fully connected triplet of nodes
cliques_of_three <- cliques(
  model, 
  size = 3, 
  threshold = 0.05 # Only consider edges with weight > 0.05
)
print(cliques_of_three)
#> Number of 3-cliques: 3 (weight threshold = 0.05)
#> Showing 3 cliques starting from clique number 1
#> 
#> Clique 1:
#>           consensus discuss emotion
#> consensus     0.082    0.19   0.073
#> discuss       0.321    0.19   0.106
#> emotion       0.320    0.10   0.077
#> 
#> Clique 2:
#>           consensus emotion plan
#> consensus     0.082   0.073 0.40
#> emotion       0.320   0.077 0.10
#> plan          0.290   0.147 0.37
#> 
#> Clique 3:
#>            consensus coregulate discuss
#> consensus      0.082      0.188    0.19
#> coregulate     0.135      0.023    0.27
#> discuss        0.321      0.084    0.19
plot(cliques_of_three, vsize = 25, edge.label.cex = 4, esize = 20, ask = FALSE)
```

```
# Identify 4-cliques (quadruples) from the TNA_Model
# A clique of size 4 means four nodes that are all mutually connected
cliques_of_four <- cliques(
  model, 
  size = 4, 
  threshold = 0.035 # Only consider edges with weight > 0.03
)
print(cliques_of_four)
#> Number of 4-cliques: 1 (weight threshold = 0.035)
#> Showing 1 cliques starting from clique number 1
#> 
#> Clique 1:
#>           consensus emotion monitor plan
#> consensus     0.082   0.073   0.047 0.40
#> emotion       0.320   0.077   0.036 0.10
#> monitor       0.159   0.091   0.018 0.22
#> plan          0.290   0.147   0.076 0.37
plot(cliques_of_four, ask = F)
```

### Centralities

#### Node-level measures

```
# Compute centrality measures for the TNA model
Centralities <- centralities(model)

# Visualize the centrality measures
plot(Centralities)
```

```
# Calculate hub scores and the authority scores for the network
hits_scores <- igraph::hits_scores(as.igraph(model))
hub_scores <- hits_scores$hub
authority_scores <- hits_scores$authority

# Print the calculated hub and authority scores for further analysis
print(hub_scores)
#>      adapt   cohesion  consensus coregulate    discuss    emotion    monitor 
#>       0.96       1.00       0.65       0.69       0.74       0.82       0.74 
#>       plan  synthesis 
#>       0.87       0.90
print(authority_scores)
#>      adapt   cohesion  consensus coregulate    discuss    emotion    monitor 
#>      0.122      0.301      1.000      0.195      0.439      0.333      0.122 
#>       plan  synthesis 
#>      0.511      0.059
```

#### Edge-level measures

```
# Edge betweenness
Edge_betweeness <- betweenness_network(model)
plot(Edge_betweeness)
```

### Community finding

```
communities <- communities(model)
print(communities)
#> Number of communities found by each algorithm:
#> 
#>         walktrap      fast_greedy       label_prop          infomap 
#>                1                3                1                1 
#> edge_betweenness    leading_eigen        spinglass 
#>                1                3                2 
#> 
#> Community assignments:
#> 
#>       state walktrap fast_greedy label_prop infomap edge_betweenness leading_eigen
#> 1     adapt        1           1          1       1                1             1
#> 2  cohesion        1           1          1       1                1             1
#> 3 consensus        1           1          1       1                1             2
#>   spinglass
#> 1         1
#> 2         1
#> 3         1
#>  [ reached 'max' / getOption("max.print") -- omitted 6 rows ]
plot(communities, method = "leading_eigen")
```

## Network inference

### Bootstrapping

```
# Perform bootstrapping on the TNA model with a fixed seed for reproducibility
set.seed(265)
boot <- bootstrap(model, threshold = 0.05)

# Print a summary of the bootstrap results
print(summary(boot))
#>    from         to weight p_value   sig ci_lower ci_upper
#> 2 adapt   cohesion 0.0029    0.51 FALSE  0.00059   0.0054
#> 3 adapt  consensus 0.0047    0.16 FALSE  0.00313   0.0065
#> 4 adapt coregulate 0.0162    0.15 FALSE  0.01078   0.0222
#> 5 adapt    discuss 0.0714    0.00  TRUE  0.06370   0.0795
#>  [ reached 'max' / getOption("max.print") -- omitted 74 rows ]

# Show the non-significant edges (p-value >= 0.05 in this case)
# These are edges that are less likely to be stable across bootstrap samples
print(boot, type = "nonsig")
#> Non-significant Edges
#> 
#>    from         to weight p_value ci_lower ci_upper
#> 2 adapt   cohesion 0.0029    0.51  0.00059   0.0054
#> 3 adapt  consensus 0.0047    0.16  0.00313   0.0065
#> 4 adapt coregulate 0.0162    0.15  0.01078   0.0222
#> 6 adapt    emotion 0.0025    0.56  0.00071   0.0044
#> 7 adapt    monitor 0.0112    0.31  0.00621   0.0172
#>  [ reached 'max' / getOption("max.print") -- omitted 22 rows ]
```

### Permutation

```
# Create TNA for the high-achievers subset (rows 1 to 1000)
Hi <- tna(group_regulation[1:1000, ])

# Create TNA for the low-achievers subset (rows 1001 to 2000)
Lo <- tna(group_regulation[1001:2000, ])

# Plot a comparison of the "Hi" and "Lo" models
# The 'minimum' parameter is set to 0.001, so edges with weights >= 0.001 are shown
plot_compare(Hi, Lo, minimum = 0.01)
```

```
# Run a permutation test to determine statistical significance of differences
# between "Hi" and "Lo"
# The 'iter' argument is set to 1000, meaning 1000 permutations are performed
Permutation <- permutation_test(Hi, Lo, iter = 1000, measures = "Betweenness")

# Plot the significant differences identified in the permutation test
plot(Permutation, minimum = 0.01)
```

```
print(Permutation$edges$stats)
#>             edge_name diff_true effect_size p_value
#> 1      adapt -> adapt   0.00000         NaN   1.000
#> 2   cohesion -> adapt   0.00533       1.991   0.060
#> 3  consensus -> adapt  -0.00132      -0.763   0.411
#> 4 coregulate -> adapt   0.01122       2.002   0.047
#> 5    discuss -> adapt  -0.09616     -11.482   0.000
#> 6    emotion -> adapt   0.00167       0.907   0.459
#> 7    monitor -> adapt  -0.00019      -0.034   0.943
#>  [ reached 'max' / getOption("max.print") -- omitted 74 rows ]
print(Permutation$centralities$stats)
#>        state  centrality diff_true effect_size p_value
#> 1      adapt Betweenness        -9       -1.31   0.220
#> 2   cohesion Betweenness         1        0.79   0.314
#> 3  consensus Betweenness         0         NaN   1.000
#> 4 coregulate Betweenness         3        1.02   0.322
#> 5    discuss Betweenness         2        4.55   0.005
#> 6    emotion Betweenness         0         NaN   1.000
#>  [ reached 'max' / getOption("max.print") -- omitted 3 rows ]
```

### Interpreting the Results of the Case-Dropping Bootstrap for Centrality Indices

```
# Results of the Case-Dropping Bootstrap for Centrality Indices
Centrality_stability <- estimate_centrality_stability(model, detailed = FALSE, iter = 100)
plot(Centrality_stability)
```
