# Supplementary material for: tna: An R Package for Transition Network Analysis
Source: Appl Psychol Meas. 2025 Jun 5;49(6):326–8. doi: 10.1177/01466216251348840 (PMC12141252; doi:10.1177/01466216251348840)
Supplement: Supplemental Material - tna: An R Package for Transition Network Analysis [file sj-zip-1-apm-10.1177_01466216251348840.zip › tna_0.4.0.tar.gz!/tna/inst/doc/grouped_sequences.html]

Using grouped sequence data with tna


# Using grouped sequence data with tna

TNA also enables the analysis of transition networks constructed from
grouped sequence data. In this example, we first fit a mixed Markov
model to the `engagement` data using the `seqHMM`
package and build a grouped TNA model based on this model. First, we
load the packages we will use for this example.

```
library("tna")
library("tibble")
library("dplyr")
library("gt")
library("seqHMM")
data("engagement", package = "tna")
```

We simulate transition probabilities to initialize the model.

```
set.seed(265)
tna_model <- tna(engagement)
n_var <- length(tna_model$labels)
n_clusters <- 3
trans_probs <- simulate_transition_probs(n_var, n_clusters)
init_probs <- list(
  c(0.70, 0.20, 0.10),
  c(0.15, 0.70, 0.15),
  c(0.10, 0.20, 0.70)
)
```

Next, we building and fit the model (this step takes some time to
compute, the final model object is also available in the
`tna` package as `engagement_mmm`).

```
mmm <- build_mmm(
  engagement,
  transition_probs = trans_probs,
  initial_probs = init_probs
)
fit_mmm <- fit_model(
  modelTrans,
  global_step = TRUE,
  control_global = list(algorithm = "NLOPT_GD_STOGO_RAND"),
  local_step = TRUE,
  threads = 60,
  control_em = list(restart = list(times = 100, n_optimum = 101))
)
```

Now, we create a new model using the cluster information from the
model. Alternatively, if sequence data is provided to
`group_model()`, the group assignments can be provided with
the `group` argument.

```
tna_model_clus <- group_model(fit_mmm$model)
```

We can summarize the cluster-specific models

```
summary(tna_model_clus) |>
  gt() |>
  fmt_number(decimals = 2)
```

| metric | Cluster 1 | Cluster 2 | Cluster 3 |
| --- | --- | --- | --- |
| Node Count | 3.00 | 3.00 | 3.00 |
| Edge Count | 8.00 | 9.00 | 9.00 |
| Network Density | 1.00 | 1.00 | 1.00 |
| Mean Distance | 0.29 | 0.30 | 0.27 |
| Mean Out-Strength | 1.00 | 1.00 | 1.00 |
| SD Out-Strength | 0.63 | 0.69 | 0.58 |
| Mean In-Strength | 1.00 | 1.00 | 1.00 |
| SD In-Strength | 0.00 | 0.00 | 0.00 |
| Mean Out-Degree | 2.67 | 3.00 | 3.00 |
| SD Out-Degree | 0.58 | 0.00 | 0.00 |
| Centralization (Out-Degree) | 0.25 | 0.00 | 0.00 |
| Centralization (In-Degree) | 0.25 | 0.00 | 0.00 |
| Reciprocity | 0.80 | 1.00 | 1.00 |

and their initial probabilities

```
bind_rows(lapply(tna_model_clus, \(x) x$inits), .id = "Cluster") |>
  gt() |>
  fmt_percent()
```

| Cluster | Active | Average | Disengaged |
| --- | --- | --- | --- |
| Cluster 1 | 75.00% | 0.00% | 25.00% |
| Cluster 2 | 51.92% | 48.08% | 0.00% |
| Cluster 3 | 0.00% | 41.07% | 58.93% |

as well as transition probabilities.

```
transitions <- lapply(
  tna_model_clus,
  function(x) {
    x$weights |>
      data.frame() |>
      rownames_to_column("From\\To") |>
      gt() |>
      tab_header(title = names(tna_model_clus)[1]) |>
      fmt_percent()
  }
)
transitions[[1]]
```

|  |  |  |  |
| --- | --- | --- | --- |
| Cluster 1 | | | |
| From\To | Active | Average | Disengaged |
| Active | 70.62% | 29.38% | 0.00% |
| Average | 51.34% | 45.98% | 2.68% |
| Disengaged | 33.33% | 38.10% | 28.57% |

```
transitions[[2]]
```

|  |  |  |  |
| --- | --- | --- | --- |
| Cluster 1 | | | |
| From\To | Active | Average | Disengaged |
| Active | 49.24% | 44.19% | 6.57% |
| Average | 32.50% | 58.61% | 8.90% |
| Disengaged | 33.33% | 57.33% | 9.33% |

```
transitions[[3]]
```

|  |  |  |  |
| --- | --- | --- | --- |
| Cluster 1 | | | |
| From\To | Active | Average | Disengaged |
| Active | 30.07% | 60.81% | 9.12% |
| Average | 15.94% | 57.32% | 26.74% |
| Disengaged | 6.58% | 47.08% | 46.35% |

We can also plot the cluster-specific transitions

```
layout(t(1:3))
plot(tna_model_clus, vsize = 20, edge.label.cex = 2)
```

Just like ordinary TNA models, we can prune the rare transitions

```
pruned_clus <- prune(tna_model_clus, threshold = 0.1)
```

and plot the cluster transitions after pruning

```
layout(t(1:3))
plot(pruned_clus, vsize = 20, edge.label.cex = 2)
```

Centrality measures can also be computed for each cluster
directly.

```
centrality_measures <- c(
  "BetweennessRSP",
  "Closeness",
  "InStrength",
  "OutStrength"
)
centralities_per_cluster <- centralities(
  tna_model_clus,
  measures = centrality_measures
)
plot(
  centralities_per_cluster, ncol = 4,
  colors = c("purple", "orange", "pink")
)
```
