# Supplementary material for: tna: An R Package for Transition Network Analysis
Source: Appl Psychol Meas. 2025 Jun 5;49(6):326–8. doi: 10.1177/01466216251348840 (PMC12141252; doi:10.1177/01466216251348840)
Supplement: Supplemental Material - tna: An R Package for Transition Network Analysis [file sj-zip-1-apm-10.1177_01466216251348840.zip › tna_0.4.0.tar.gz!/tna/inst/doc/communities_and_cliques.html]

Finding cliques and communities with tna


# Finding cliques and communities with tna

The `tna` package includes functionalities for finding
cliques of the transition network as well as discovering communities. We
begin by loading the package and the example data set
`group_regulation`.

```
library("tna")
data("group_regulation", package = "tna")
```

We fit the TNA model to the data.

```
tna_model <- tna(group_regulation)
print(tna_model)
#> State Labels
#> 
#> adapt, cohesion, consensus, coregulate, discuss, emotion, monitor, plan, synthesis 
#> 
#> Transition Probability Matrix
#> 
#>                   adapt   cohesion  consensus coregulate    discuss    emotion
#> adapt      0.0000000000 0.27308448 0.47740668 0.02161100 0.05893910 0.11984283
#> cohesion   0.0029498525 0.02713864 0.49793510 0.11917404 0.05958702 0.11563422
#> consensus  0.0047400853 0.01485227 0.08200348 0.18770738 0.18802338 0.07268131
#> coregulate 0.0162436548 0.03604061 0.13451777 0.02335025 0.27360406 0.17208122
#> discuss    0.0713743356 0.04758289 0.32118451 0.08428246 0.19488737 0.10579600
#> emotion    0.0024673951 0.32534367 0.32040888 0.03419105 0.10186817 0.07684173
#> monitor    0.0111653873 0.05582694 0.15910677 0.05792045 0.37543615 0.09071877
#> plan       0.0009745006 0.02517460 0.29040117 0.01721618 0.06789021 0.14682475
#> synthesis  0.2346625767 0.03374233 0.46625767 0.04447853 0.06288344 0.07055215
#>               monitor       plan   synthesis
#> adapt      0.03339882 0.01571709 0.000000000
#> cohesion   0.03303835 0.14100295 0.003539823
#> consensus  0.04661084 0.39579712 0.007584137
#> coregulate 0.08629442 0.23908629 0.018781726
#> discuss    0.02227284 0.01164262 0.140976968
#> emotion    0.03630596 0.09975326 0.002819880
#> monitor    0.01814375 0.21563154 0.016050244
#> plan       0.07552379 0.37420822 0.001786584
#> synthesis  0.01226994 0.07515337 0.000000000
#> 
#> Initial Probabilities
#> 
#>      adapt   cohesion  consensus coregulate    discuss    emotion    monitor 
#>     0.0115     0.0605     0.2140     0.0190     0.1755     0.1515     0.1440 
#>       plan  synthesis 
#>     0.2045     0.0195
plot(tna_model)
```

Next, we apply several community finding algorithms to the model (see
`?communities` for more details), and plot the results for
the `leading_eigen` algorithm.

```
cd <- communities(tna_model)
plot(cd, method = "leading_eigen")
```

Cliques can be obtained with the `cliques` function. Here
we look for dyads and triads by setting `size = 2` and
`size = 3`, respectively. Finally, we plot the results.

```
layout(matrix(1:4, ncol = 2, byrow = T))
dyads <- cliques(tna_model, size = 2, threshold = 0.2)
triads <- cliques(tna_model, size = 3, threshold = 0.05)
plot(dyads, ask = F)
plot(triads, ask = F)
```
