# Supplementary material for: tna: An R Package for Transition Network Analysis
Source: Appl Psychol Meas. 2025 Jun 5;49(6):326–8. doi: 10.1177/01466216251348840 (PMC12141252; doi:10.1177/01466216251348840)
Supplement: Supplemental Material - tna: An R Package for Transition Network Analysis [file sj-zip-1-apm-10.1177_01466216251348840.zip › tna_0.4.0.tar.gz!/tna/inst/doc/prepare_data.html]

How to prepare data for tna


# How to prepare data for tna

The `tna` package accepts sequence data (a
`stslist` object created by the `TraMineR`
package), a wide `data.frame` where each row represents a
sequence and each column represents a timepoint, or a transition matrix.
When our data does not follow any of those formats, we can use the
`prepare_data` function from `tna` to get it in
the right shape.

Let’s start by creating an example dataframe containing event logs
where each row represents an action performed by a `user` at
a specific `timestamp.` The `achievement` column
indicates whether the user was associated with high or low
achievement.

```
df <- tribble(
  ~user, ~timestamp, ~event, ~achievement, ~order,
  2, "2025-02-27 18:01:32", "Plan",           "High", 1,
  2, "2025-02-27 18:03:32", "Goals",          "High", 2,
  1, "2025-02-27 18:08:32", "Goals",          "High", 1,
  3, "2025-02-27 18:15:32", "Plan",           "High", 1,
  5, "2025-02-27 18:16:32", "Help",           "Low",  1,
  3, "2025-02-27 18:19:32", "Goals",          "High", 2,
  5, "2025-02-27 18:19:32", "Plan",           "Low",  2,
  2, "2025-02-27 18:20:32", "Environment",    "High", 3,
  1, "2025-02-27 18:25:32", "Task",           "High", 2,
  4, "2025-02-27 18:25:32", "Help",           "Low",  1,
  5, "2025-02-27 18:26:32", "Task",           "Low",  3,
  3, "2025-02-27 18:32:32", "Metacognition",  "High", 3,
  4, "2025-02-27 18:33:32", "Goals",          "Low",  2,
  1, "2025-02-27 18:36:32", "Environment",    "High", 3,
  1, "2025-02-27 18:44:32", "Task",           "High", 4,
  1, "2025-02-27 18:45:32", "Task",           "High", 5,
  5, "2025-02-27 18:46:32", "Help",           "Low",  4,
  1, "2025-02-27 19:01:32", "Plan",           "High", 6,
  2, "2025-02-27 19:06:32", "Environment",    "High", 4,
  4, "2025-02-27 19:06:32", "Plan",           "Low",  3,
  1, "2025-02-27 19:13:32", "Metacognition",  "High", 7,
  3, "2025-02-27 19:13:32", "Metacognition",  "High", 4,
  4, "2025-02-27 19:15:32", "Goals",          "Low",  4,
  4, "2025-02-27 19:20:32", "Metacognition",  "Low",  5,
  4, "2025-02-27 19:20:32", "Environment",    "Low",  6,
  5, "2025-02-27 19:23:32", "Metacognition",  "Low",  5,
  3, "2025-02-27 19:25:32", "Help",           "High", 5,
  2, "2025-02-27 19:27:32", "Metacognition",  "High", 5,
  2, "2025-02-27 19:33:32", "Environment",    "High", 6,
  4, "2025-02-27 19:46:32", "Environment",    "Low",  7,
  5, "2025-02-27 19:49:32", "Plan",           "Low",  6,
  2, "2025-02-27 19:55:32", "Goals",          "High", 7
)
```

When we are interested in the whole dataset as a single sequence, and
the data is already ordered chronologically, we just need to specify the
`action` column, which in this case is called
`event`. We can pass the result of calling
`prepare_data` directly to the `tna` function to
create a `tna` model.

```
by_classroom <- prepare_data(df, action = "event")
tna_by_classroom <- tna(by_classroom)
plot(tna_by_classroom)
```

When we want to create a sequence per user, in addition to
`action`, we need to specify the `actor` column,
which in this case is `user`. If the data is already ordered,
we do not need to provide any additional arguments. If it is not
ordered, we can provide a column to order the data by, in this case
`order`.

```
by_user <- prepare_data(df, actor = "user", action = "event", order = "order")
tna_by_user <- tna(by_user)
plot(tna_by_user)
```

If rather than the order we only have the timestamps, we can provide
it as `time` column. By default, events happening less than
15 minutes apart will be grouped in the same sequence, while events that
happen after a longer time, will mark the start of a new sequence (i.e.,
session). If both `time` and `order` are provided,
the data will be first ordered by `time`, and in case of a
tie, by `order`.

```
by_session <- prepare_data(df, actor = "user", time = "timestamp", action = "event")
tna_by_session <- tna(by_session)
plot(tna_by_session)
```

If we want to customize the time gap that marks the start of a new
sequence, we can do so by customizing the `time_threshold`
argument (in minutes).

```
by_session_custom <- prepare_data(df, actor = "user", time = "timestamp", action = "event", 
                                  time_threshold = 10*60) # 10 minutes
tna_by_session_custom <- tna(by_session_custom)
plot(tna_by_session_custom)
```

Another advantage of using `prepare_data` prior to
constructing the `tna` model is that we get to keep other
variables of the data and use them in our analysis. For instance, we can
use `group_tna` to create a `tna` model by
achievement group just by passing the result of
`prepare_data` as a first argument, and indicating the name
of the column in the data that we want to group by.

```
gtna <- group_tna(by_user, group = "achievement")
plot(gtna)
```
