# Supplementary material for: tna: An R Package for Transition Network Analysis
Source: Appl Psychol Meas. 2025 Jun 5;49(6):326–8. doi: 10.1177/01466216251348840 (PMC12141252; doi:10.1177/01466216251348840)
Supplement: Supplemental Material - tna: An R Package for Transition Network Analysis [file sj-zip-1-apm-10.1177_01466216251348840.zip › tna_0.4.0.tar.gz!/tna/inst/doc/ftna.html]

Frequency-based tna


# Frequency-based tna

# FTNA tutorial

```
# Install 'tna' package from CRAN if needed (uncomment if required).
# install.packages("tna")

# Load packages
library("tna")

# Load example data provided within the 'tna' package, 
# representing group regulatory interactions
data(group_regulation)

# Run FTNA on 'group_regulation' data using raw counts of 
# transitions ("absolute" type) and print the result
model <- ftna(group_regulation)

# Print the output to inspect the model
print(model)
#> State Labels
#> 
#> adapt, cohesion, consensus, coregulate, discuss, emotion, monitor, plan, synthesis 
#> 
#> Transition Frequency Matrix
#> 
#>            adapt cohesion consensus coregulate discuss emotion monitor plan
#> adapt          0      139       243         11      30      61      17    8
#> cohesion       5       46       844        202     101     196      56  239
#> consensus     30       94       519       1188    1190     460     295 2505
#>            synthesis
#> adapt              0
#> cohesion           6
#> consensus         48
#>  [ reached getOption("max.print") -- omitted 6 rows ]
#> 
#> Initial Probabilities
#> 
#>      adapt   cohesion  consensus coregulate    discuss    emotion    monitor 
#>      0.011      0.060      0.214      0.019      0.175      0.151      0.144 
#>       plan  synthesis 
#>      0.204      0.019
```

```
# Calculate the Transition Network Analysis (TNA) on the group_regulation 
# data with scaled weights between 0 and 1
model_scaled <- ftna(group_regulation, scaling = "minmax")
print(model_scaled) # Print the FTNA model with scaled weights
#> State Labels
#> 
#> adapt, cohesion, consensus, coregulate, discuss, emotion, monitor, plan, synthesis 
#> 
#> Transition Frequency Matrix
#> 
#>             adapt cohesion consensus coregulate discuss emotion monitor   plan
#> adapt      0.0000   0.0555     0.097     0.0044   0.012   0.024  0.0068 0.0032
#> cohesion   0.0020   0.0184     0.337     0.0806   0.040   0.078  0.0224 0.0954
#> consensus  0.0120   0.0375     0.207     0.4743   0.475   0.184  0.1178 1.0000
#>            synthesis
#> adapt         0.0000
#> cohesion      0.0024
#> consensus     0.0192
#>  [ reached getOption("max.print") -- omitted 6 rows ]
#> 
#> Initial Probabilities
#> 
#>      adapt   cohesion  consensus coregulate    discuss    emotion    monitor 
#>      0.011      0.060      0.214      0.019      0.175      0.151      0.144 
#>       plan  synthesis 
#>      0.204      0.019
```

## Plotting

```
# Plotting the two weights together to see if the scaling distorts the data

# Combine weights from absolute and scaled models into a data frame for plotting
weights_data <- data.frame(
  Absolute = as.vector(model$weights), # Extract absolute weights as a vector
  Scaled = as.vector(model_scaled$weights) # Extract scaled weights as a vector
)
corr <- cor(weights_data$Absolute, weights_data$Scaled, method = c("pearson")) |>
  round(digits = 2)

# Create a scatter plot comparing absolute vs. scaled weights
plot_abs_scaled <- ggplot(weights_data, aes(x = Absolute, y = Scaled)) +
  geom_point(color = "steelblue", alpha = 0.6, size = 2) +  # Add points with specified aesthetics
  geom_smooth(formula = y ~ x, method = "lm", color = "red", linetype = "dashed") + # Add a linear trend line
  # geom_smooth(method = lm, formula = y ~ x, se = FALSE) +
  geom_text(x = 0.1, y = 0.9, label = paste0('r = ', corr), color = 'red')
  # stat_cor(aes(label = after_stat(r.label)), label.x = 0.1, label.y = 0.9, 
  #          size = 4, color = "black", method = "spearman") + # Display Spearman correlation
  labs(x = "Absolute Weights", y = "Scaled Weights") + # Label axes
  theme_minimal() # Apply a minimal theme for the plot
#> NULL

# Display the scatter plot
plot_abs_scaled
```

```
# Calculate the Transition Network Analysis (TNA) on the `group_regulation` 
# data with ranked weights
model_ranked <- ftna(group_regulation, scaling = "rank")
print(model_ranked) # Print the FTNA model with ranked weights
```

```
# Combine weights from absolute and ranked models into a data frame for plotting
weights_data <- data.frame(
  Absolute = as.vector(model$weights), # Extract absolute weights as a vector
  Ranked = as.vector(model_ranked$weights) # Extract ranked weights as a vector
)

# Create a scatter plot comparing Absolute vs. Ranked weights with correlation annotations
plot_abs_ranked <- ggplot(weights_data, aes(x = Absolute, y = Ranked)) +
  geom_point(color = "steelblue", alpha = 0.6, size = 2) +  # Add points with specified aesthetics
  geom_smooth(formula = y ~ x, method = "lm", color = "red", linetype = "dashed") +  # Add a linear trend line
  # stat_cor(aes(label = paste("Spearman: ", round(after_stat(r), 2))), 
  #          method = "spearman", label.x = 0.1, label.y = 0.9, size = 4, color = "black") + # Spearman correlation annotation
  # stat_cor(aes(label = paste("Pearson: ", round(after_stat(r), 2))), 
  #          method = "pearson", label.x = 0.1, label.y = 0.8, size = 4, color = "darkgreen") + # Pearson correlation annotation
  labs(x = "Absolute Weights", y = "Ranked Weights") + # Label axes
  theme_minimal() # Apply a minimal theme for the plot

# Display the scatter plot
plot_abs_ranked
```

## Pruning

```
layout(matrix(1:4, ncol = 2))
# Pruning with different methods
pruned_threshold <- prune(model_scaled, method = "threshold", threshold = 0.1)
pruned_lowest <- prune(model_scaled, method = "lowest", lowest = 0.15)
pruned_disparity <- prune(model_scaled, method = "disparity", alpha = 0.5)

# Plotting for comparison
plot(pruned_threshold)
plot(pruned_lowest)
plot(pruned_disparity)
plot(model_scaled, minimum = 0.05, cut = 0.1)
```

## Patterns

```
# Identify 2-cliques (dyads) from the FTNA model with a weight threshold, 
# excluding loops in visualization.
# A 2-clique represents a pair of nodes that are strongly connected based on 
# the specified weight threshold.
layout(matrix(1:6, ncol = 3))
cliques_of_two <- cliques(
  model_scaled,      # The FTNA model with scaled edge weights
  size = 2,          # Looking for pairs of connected nodes (dyads)
  threshold = 0.1    # Only include edges with weights greater than 0.1
)

# Print and visualize the identified 2-cliques (dyads)
print(cliques_of_two)  # Display details of 2-cliques
#> Number of 2-cliques: 8 (weight threshold = 0.1)
#> Showing 6 cliques starting from clique number 1
#> 
#> Clique 1:
#>           consensus plan
#> consensus      0.21 1.00
#> plan           0.71 0.92
#> 
#> Clique 2:
#>           consensus discuss
#> consensus      0.21    0.48
#> discuss        0.51    0.31
#> 
#> Clique 3:
#>         discuss emotion
#> discuss    0.31   0.167
#> emotion    0.12   0.087
#> 
#> Clique 4:
#>         emotion plan
#> emotion   0.087 0.11
#> plan      0.361 0.92
#> 
#> Clique 5:
#>           consensus emotion
#> consensus      0.21   0.184
#> emotion        0.36   0.087
#> 
#> Clique 6:
#>         monitor plan
#> monitor    0.01 0.12
#> plan       0.19 0.92
plot(cliques_of_two, ask = F, vsize = 20)   # Visualize 2-cliques in the network
```

```
layout(matrix(1:6, ncol = 3))
# Identify 3-cliques (triads) from the FTNA model.
# A 3-clique is a fully connected set of three nodes, indicating a strong 
# triplet structure.
cliques_of_three <- cliques(
  model_scaled,      # The FTNA model with scaled edge weights
  size = 3,          # Looking for triplets of fully connected nodes (triads)
  threshold = 0.05   # Only include edges with weights greater than 0.05
)

# Print and visualize the identified 3-cliques (triads)
# Uncomment the code below to view the results
print(cliques_of_three) # Display details of 3-cliques
#> Number of 3-cliques: 5 (weight threshold = 0.05)
#> Showing 5 cliques starting from clique number 1
#> 
#> Clique 1:
#>            consensus coregulate discuss
#> consensus       0.21      0.474    0.48
#> coregulate      0.11      0.018    0.22
#> discuss         0.51      0.133    0.31
#> 
#> Clique 2:
#>           consensus discuss emotion
#> consensus      0.21    0.48   0.184
#> discuss        0.51    0.31   0.167
#> emotion        0.36    0.12   0.087
#> 
#> Clique 3:
#>           consensus emotion plan
#> consensus      0.21   0.184 1.00
#> emotion        0.36   0.087 0.11
#> plan           0.71   0.361 0.92
#> 
#> Clique 4:
#>           consensus monitor plan
#> consensus     0.207    0.12 1.00
#> monitor       0.091    0.01 0.12
#> plan          0.714    0.19 0.92
#> 
#> Clique 5:
#>          cohesion emotion  plan
#> cohesion    0.018   0.078 0.095
#> emotion     0.368   0.087 0.113
#> plan        0.062   0.361 0.920
plot(cliques_of_three, ask = F)  # Visualize 3-cliques in the network
```

```
layout(matrix(1:6, ncol = 3))
# Identify 4-cliques (quadruples) from the FTNA model.
# A 4-clique includes four nodes where each node is connected to every other 
# node in the group.
# Uncomment the code below to view the results
cliques_of_four <- cliques(
  model_scaled,      # The FTNA model with scaled edge weights
  size = 4,          # Looking for quadruples of fully connected nodes (4-cliques)
  threshold = 0.03   # Only include edges with weights greater than 0.03
)

# Print and visualize the identified 4-cliques (quadruples) 
# Uncomment the code below to view the results
print(cliques_of_four)  # Display details of 4-cliques
#> Number of 4-cliques: 11 (weight threshold = 0.03)
#> Showing 6 cliques starting from clique number 1
#> 
#> Clique 1:
#>            consensus coregulate emotion plan
#> consensus       0.21      0.474   0.184 1.00
#> coregulate      0.11      0.018   0.135 0.19
#> emotion         0.36      0.039   0.087 0.11
#> plan            0.71      0.042   0.361 0.92
#> 
#> Clique 2:
#>            consensus coregulate discuss emotion
#> consensus       0.21      0.474    0.48   0.184
#> coregulate      0.11      0.018    0.22   0.135
#> discuss         0.51      0.133    0.31   0.167
#> emotion         0.36      0.039    0.12   0.087
#> 
#> Clique 3:
#>            coregulate discuss emotion monitor
#> coregulate      0.018    0.22   0.135   0.068
#> discuss         0.133    0.31   0.167   0.035
#> emotion         0.039    0.12   0.087   0.041
#> monitor         0.033    0.21   0.052   0.010
#> 
#> Clique 4:
#>            consensus coregulate discuss monitor
#> consensus      0.207      0.474    0.48   0.118
#> coregulate     0.106      0.018    0.22   0.068
#> discuss        0.507      0.133    0.31   0.035
#> monitor        0.091      0.033    0.21   0.010
#> 
#> Clique 5:
#>           consensus discuss emotion monitor
#> consensus     0.207    0.48   0.184   0.118
#> discuss       0.507    0.31   0.167   0.035
#> emotion       0.363    0.12   0.087   0.041
#> monitor       0.091    0.21   0.052   0.010
#> 
#> Clique 6:
#>            coregulate emotion monitor plan
#> coregulate      0.018   0.135   0.068 0.19
#> emotion         0.039   0.087   0.041 0.11
#> monitor         0.033   0.052   0.010 0.12
#> plan            0.042   0.361   0.186 0.92
plot(cliques_of_four, ask = F)   # Visualize 4-cliques in the network
```

```
# Identify 5-cliques (quintuples) from the FTNA model, summing edge weights.
# Here, the sum of edge weights in both directions must meet the specified 
# threshold for inclusion.
# Uncomment the code below to view the results
cliques_of_five <- cliques(
  model_scaled,      # The FTNA model with scaled edge weights
  size = 5,          # Looking for quintuples of fully connected nodes (5-cliques)
  threshold = 0.1,   # Only edges with total bidirectional weights greater than 0.1
  sum_weights = TRUE # Sum edge weight in both directions when computing  threshold
)

# Print and visualize the identified 5-cliques (quintuples)
print(cliques_of_five)  # Display details of 5-cliques
#> Number of 5-cliques: 1 (weight threshold = 0.1)
#> Showing 1 cliques starting from clique number 1
#> 
#> Clique 1:
#>            consensus coregulate discuss emotion  plan
#> consensus       0.21      0.474    0.48   0.184 1.000
#> coregulate      0.11      0.018    0.22   0.135 0.188
#> discuss         0.51      0.133    0.31   0.167 0.018
#> emotion         0.36      0.039    0.12   0.087 0.113
#> plan            0.71      0.042    0.17   0.361 0.920
plot(cliques_of_five, ask = F)   # Visualize 5-cliques in the network
```

## Graph level measures

```
summary(model_scaled)
#> # A tibble: 13 × 2
#>    metric                        value
#>  * <chr>                         <dbl>
#>  1 Node Count                   9     
#>  2 Edge Count                  78     
#>  3 Network Density              1     
#>  4 Mean Distance                0.0240
#>  5 Mean Out-Strength            1.13  
#>  6 SD Out-Strength              0.886 
#>  7 Mean In-Strength             1.13  
#>  8 SD In-Strength               0.878 
#>  9 Mean Out-Degree              8.67  
#> 10 SD Out-Degree                0.707 
#> 11 Centralization (Out-Degree)  0.0156
#> 12 Centralization (In-Degree)   0.0156
#> 13 Reciprocity                  0.986
```

```
summary(pruned_disparity)
#> # A tibble: 13 × 2
#>    metric                       value
#>  * <chr>                        <dbl>
#>  1 Node Count                   9    
#>  2 Edge Count                  39    
#>  3 Network Density              0.542
#>  4 Mean Distance                0.188
#>  5 Mean Out-Strength            0.900
#>  6 SD Out-Strength              0.700
#>  7 Mean In-Strength             0.900
#>  8 SD In-Strength               0.673
#>  9 Mean Out-Degree              4.33 
#> 10 SD Out-Degree                1.22 
#> 11 Centralization (Out-Degree)  0.234
#> 12 Centralization (In-Degree)   0.516
#> 13 Reciprocity                  0.615
```

## Node level measures

```
# Compute centrality measures for the FTNA model
centrality_measures <- centralities(model_scaled)

# Print the calculated centrality measures in the FTNA model
print(centrality_measures)
#> # A tibble: 9 × 10
#>   state      OutStrength InStrength ClosenessIn ClosenessOut Closeness Betweenness
#> * <fct>            <dbl>      <dbl>       <dbl>        <dbl>     <dbl>       <dbl>
#> 1 adapt            0.203      0.212       14.6          5.83     21.6           20
#> 2 cohesion         0.658      0.667        6.61         6.54     18.7            0
#> 3 consensus        2.32       2.34         1.25         5.89      7.68           0
#> 4 coregulate       0.768      0.818       11.3          4.02     13.0            0
#> 5 discuss          1.27       1.26         7.22         3.30      7.68           0
#> 6 emotion          1.05       1.02         4.46         6.68     16.9            0
#> 7 monitor          0.562      0.480       10.8          5.24     13.0            7
#> 8 plan             1.54       1.56        11.6          5.88     16.8            9
#> 9 synthesis        0.260      0.275       12.0          5.72     16.9           21
#> # ℹ 3 more variables: BetweennessRSP <dbl>, Diffusion <dbl>, Clustering <dbl>
plot(centrality_measures)
```

```
# Convert the FTNA model to an igraph object and 
# calculate HITS (Hub and Authority) scores
hits_results <- igraph::hits_scores(as.igraph(model_scaled))

# Extract the hub and authority scores from the HITS results for further analysis
hub_scores <- hits_results$hub
authority_scores <- hits_results$authority
```

```
# Print the hub and authority scores to view influential nodes
print(hub_scores)
#>      adapt   cohesion  consensus coregulate    discuss    emotion    monitor 
#>      0.056      0.240      0.955      0.256      0.377      0.305      0.189 
#>       plan  synthesis 
#>      1.000      0.074
print(authority_scores)
#>      adapt   cohesion  consensus coregulate    discuss    emotion    monitor 
#>      0.033      0.129      0.672      0.293      0.437      0.344      0.174 
#>       plan  synthesis 
#>      1.000      0.056
```

## Edge level measures

```
edge_between <- betweenness_network(model_scaled)
plot(edge_between)
```

## Community detection

```
detected_communities <- communities(model_scaled)
plot(detected_communities, minimum = 0.05)
```

```
print(detected_communities)
#> Number of communities found by each algorithm:
#> 
#>         walktrap      fast_greedy       label_prop          infomap 
#>                1                3                1                1 
#> edge_betweenness    leading_eigen        spinglass 
#>                1                3                4 
#> 
#> Community assignments:
#> 
#>       state walktrap fast_greedy label_prop infomap edge_betweenness leading_eigen
#> 1     adapt        1           1          1       1                1             1
#> 2  cohesion        1           2          1       1                1             3
#> 3 consensus        1           3          1       1                1             2
#>   spinglass
#> 1         2
#> 2         4
#> 3         1
#>  [ reached 'max' / getOption("max.print") -- omitted 6 rows ]
```

## Bootstrapping

```
# Perform bootstrapping on the FTNA model with a fixed seed for reproducibility
set.seed(265)
boot <- bootstrap(model_scaled, threshold = 0.05)

# Print the combined results data frame containing
print(summary(boot))
#>    from         to weight p_value   sig ci_lower ci_upper
#> 2 adapt   cohesion  0.002    0.51 FALSE   0.0004   0.0036
#> 3 adapt  consensus  0.012    0.16 FALSE   0.0078   0.0163
#> 4 adapt coregulate  0.013    0.16 FALSE   0.0085   0.0177
#> 5 adapt    discuss  0.113    0.00  TRUE   0.0998   0.1269
#>  [ reached 'max' / getOption("max.print") -- omitted 74 rows ]

# View non-significant edges  which are less likely to be stable across bootstrap samples
print(boot, type = "nonsig")
#> Non-significant Edges
#> 
#>    from         to weight p_value ci_lower ci_upper
#> 2 adapt   cohesion 0.0020    0.51  0.00040   0.0036
#> 3 adapt  consensus 0.0120    0.16  0.00782   0.0163
#> 4 adapt coregulate 0.0128    0.16  0.00850   0.0177
#> 6 adapt    emotion 0.0028    0.56  0.00079   0.0049
#> 7 adapt    monitor 0.0064    0.33  0.00355   0.0099
#>  [ reached 'max' / getOption("max.print") -- omitted 23 rows ]
```

## Comparing Models

```
# Create FTNA for the high-achievers subset (rows 1 to 1000)
Hi <- ftna(group_regulation[1:1000, ], scaling = "minmax")

# Create FTNA for the low-achievers subset (rows 1001 to 2000)
Lo <- ftna(group_regulation[1001:2000, ], scaling = "minmax")

# Plot a comparison of the "Hi" and "Lo" models
# The 'minimum' parameter is set to 0.001, so edges with weights >= 0.001 are shown
plot_compare(Hi, Lo, minimum = 0.0001)
```

```
# Run a permutation test to determine statistical significance of 
# differences between "Hi" and "Lo"
# The 'it' parameter is set to 1000, meaning 1000 permutations are performed
Permutation <- permutation_test(Hi, Lo, it = 1000)

# Plot the significant differences identified in the permutation test
plot(Permutation, minimum = 0.01)
```

## Centrality stability

```
Centrality_stability <- estimate_centrality_stability(model_scaled, detailed = FALSE, iter = 100)
plot(Centrality_stability)
```
